# Dramatic tumour response to pemetrexed single-agent in an elderly patient with malignant peritoneal mesothelioma: a case report

**DOI:** 10.1186/1471-2407-6-289

**Published:** 2006-12-18

**Authors:** Gianpiero Fasola, Fabio Puglisi, Alessandro Follador, Marianna Aita, Silvia Di Terlizzi, Ornella Belvedere

**Affiliations:** 1 Dept. of Medical Oncology, University Hospital, P.le S. M. Misericordia, 33100 Udine, Italy

## Abstract

**Background:**

To date, there is no standard treatment for unresectable malignant peritoneal mesothelioma; either best supportive care or systemic chemotherapy with palliative intent are accepted options.

**Case presentation:**

Here, we report the case of a 79-year old patient with malignant peritoneal mesothelioma who was treated with pemetrexed single-agent and obtained an impressive long-lasting response.

**Conclusion:**

Single-agent pemetrexed is a treatment option for malignant peritoneal mesothelioma in selected elderly patients or in patients with unpaired performance status.

## Background

Malignant peritoneal mesothelioma (MPM) accounts for 10–30% of all malignant mesothelioma cases [1]. Although a direct causal relationship between pleural mesothelioma and occupational asbestos exposure is strong [2], only 33% of patients with MPM report a history of asbestos exposure [3]. Most of the patients present with abdominal distension and/or pain, and occasionally bowel obstruction; other signs at presentation include ascites, tenderness, and palpable masses [2]. Findings at computed tomography (CT) scan may appear similar to other metastatic tumours, with nodules, plaques, thickening, masses involving the parietal and visceral peritoneum, and lymphadenopathy. A definite diagnosis of MPM can be difficult and requires histological examination with appropriate immunohistochemical analysis on adequate biopsy samples. Malignant mesothelioma is characterized by the presence of staining for calretinin, cytokeratin 5/6, and absence of staining for carcinoembryonic antigen and MOC-31 (or B72.3, Ber-EP4, or BG-8) [4].

Prognosis of MPM is dismal and there is no standard treatment approach: although multimodality treatment including intraperitoneal chemotherapy and cytoreductive surgery may result in prolonged survival in selected patients [5], either best supportive care or systemic chemotherapy with palliative intent are accepted approaches.

Here we report a case of an elderly patient with unresectable MPM treated with single-agent pemetrexed.

## Case presentation

In April 2004, a 79-year-old man presented with a 4 month history of subcutaneous abdominal nodules and bilateral inguinal lymphadenopathy; no other signs or symptoms were present.

The patient was retired after various occupations; he had also worked for 7 years at the local port and for 3 years in a motor factory, but he had no memory of direct exposure to asbestos. He had no smoking history. Medical history included partial gastric resection due to gastric and duodenal ulcers with concomitant cholecystectomy for cholelithiasis at 44, surgical removal of a renal stone at 74, diagnosis of benign prostatic hypertrophy at 76.

A CT of the chest, abdomen and pelvis showed the presence of a large mass occupying the epigastrium and the left upper region of the abdomen (longest diameter: 9 cm), and multiple diffuse abdominal nodules along the peritoneal surface and below the anterior abdominal wall (longest diameter: 5 cm); imaging confirmed the presence of bilateral inguinal lymphadenopathy. A small amount of ascites was present; liver, spleen and kidneys were spared. Chest CT was negative, with no evidence of pleural or pulmonary parenchymal abnormalities. An excisional biopsy of the left inguinal lymphadenopathy was performed. Histological analysis showed malignant neoplasm with a micropapillary, microcystic, and solid growth pattern infiltrating the adjacent soft tissues. Immunohistochemical analysis revealed positive staining of tumour cells for cytokeratins 5 and 7, vimentin, epithelial membrane antigen (EMA), epithelial specific antigen (ESA), calretinin; no reactivity was observed for carcinoembryonic antigen or Tag/B72.3. Histological and immunohistochemical findings were consistent with a well differentiated biphasic malignancy of mesothelial origin.

In July 2004 the patient was referred to our Department for management. At admission, he reported progressive clinical deterioration (Karnofsky Performance Status 80), fatigue, unintentional weight loss of 6 kg and persistent fever over the last few weeks. His medications were terazosine and finasteride. Physical examination showed a subcutaneous epi-mesogastric mass (9 × 6 cm) and bilateral inguinal lymphadenopathy. Complete blood count showed haemoglobin 11.6 g/dL, platelet count 559,000 per cubic millimeter, and white cell count 8,400 per cubic millimeter, with a normal differential count; renal function and liver function tests were normal.

**Figure 1:**
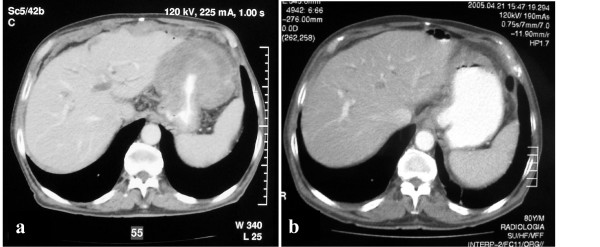
Axial contrast-enhanced CT scan at diagnosis (a) showing a heterogeneous mass encasing the stomach in the upper left quadrant, and diffuse multiple omental nodules; (b) axial contrast-enhanced CT scan after 8 cycles of pemetrexed showing a partial response with a significant shrinkage of the tumour mass and disappearance of the omental nodules.

In consideration of patient's age, and type and stage of disease, palliative chemotherapy with single-agent pemetrexed at 500 mg/m^2 ^i.v. every 3 weeks was proposed [6]. Pemetrexed was provided by Eli Lilly (Indianapolis, IN) within an Expanded Access Programme. Before chemotherapy start, a written informed consent was obtained and a repeat CT was performed, showing a size increase of all lesions (longest diameter of the mass in the abdominal left upper region: 12 cm) (Fig. 1a). Treatment started in early August 2004. During the treatment, the patient received vitamin B12 and folic acid supplementation, and steroid prophylaxis [6]. After the first 2 cycles, a clinical improvement was observed with reduction of frequency and intensity of febrile episodes. Partial response was documented by physical examination (decrease in size of the subcutaneous abdominal mass and bilateral inguinal nodes) and by CT (decrease in size of all known lesions and no new lesions). Treatment was well tolerated with only grade 2 neutropenia, and grade 2 nausea and anorexia. Therefore, pemetrexed was continued for 3 cycles and further tumour shrinkage was documented at physical examination and imaging. Toxicity persisted acceptable with only grade 2 nausea and fatigue; fever disappeared after the third cycle. Three further cycles of pemetrexed were administered without increase in toxicity except for mild fever lasting few days after drug administration. After a total of 8 cycles, the epi-mesogastric mass and the inguinal bilateral lymphadenopathy were no more detectable at physical examination, whereas abdominal CT in April 2005 showed a minimal residue of disease (Fig. 1b). His Karnofsky Performance Status was 80 and his weight was increased of approximately 10% since treatment start. At that point, after multidisciplinary evaluation and discussion with the patient about therapeutic options, we decided to stop the treatment.

In August 2005, a follow-up CT revealed the reappearance of nodular peritoneal lesions. Due to the significant benefit observed and the duration of response (11 months) the patient was retreated with pemetrexed for 4 cycles from August to November 2005, resulting in disease stabilization. At the time of his last appointment at our Department in February 2006, a CT scan confirmed stable disease.

## Discussion

The efficacy of systemic chemotherapy as well as chemotherapy regimens to be used in patients with MPM are mainly extrapolated from studies in patients with malignant pleural mesothelioma. In a randomized phase III trial in malignant pleural mesothelioma, pemetrexed, a novel multitargeted antifolate, showed significantly longer survival in combination with cisplatin compared to cisplatin alone (median survival 12.1 vs. 9.3 months; p < 0.02) [7].

A subset analysis of data from patients with MPM in an expanded access program showed a favourable safety profile and encouraging activity for pemetrexed alone or in combination with cisplatin, both in the first- and in the second-line setting [8]. Disease control rate (complete response + partial response + stable disease) was 71%; overall response rate was 25% and 23% in chemonaive (n = 28) and previously treated (n = 43) patients, respectively. The most common adverse events (i.e. dehydration, nausea, vomiting) were clinically manageable. In that series, only 3 chemotherapy-naive patients who received pemetrexed single agent were included: no response was observed (2 stable disease, 1 progressive disease). Here, we report durable disease control in an elderly chemo-naive patient with MPM, obtained with pemetrexed single agent, without significant toxicity.

Due to the favourable toxicity profile and activity in MPM, pemetrexed single-agent may be a treatment option for selected elderly patients and patients unfit for a platinum based chemotherapy. A prospective clinical trial should be conducted to confirm this indication. However, MPM is a rare tumour; large trials evaluating new treatment approaches in this setting, especially in selected patients (i.e. elderly or poor performance status patients), are in general not feasible.

## Conclusion

Although the use of pemetrexed in malignant mesothelioma is mostly supported in combination with platinum, our report suggests that single-agent pemetrexed may be considered for selected elderly or unfit patients with MPM.

## Competing interests

The author(s) declare that they have no competing interests.

## Authors' contributions

GF, AF, OB and SDT have made substantial contributions to conception of the work and acquisition of data.

GF, OB, FP and MA have been involved in drafting the manuscript and revising it for intellectual content.

All Authors have given approval to the final version of the work.

## Pre-publication history

The pre-publication history for this paper can be accessed here:
